# Supplementary figures and images for: Spoink, a LTR retrotransposon, invaded D. melanogaster populations in the 1990s
Source: PLoS Genet. 2024 Mar 26;20(3):e1011201. doi: 10.1371/journal.pgen.1011201 (PMC10965091; doi:10.1371/journal.pgen.1011201)

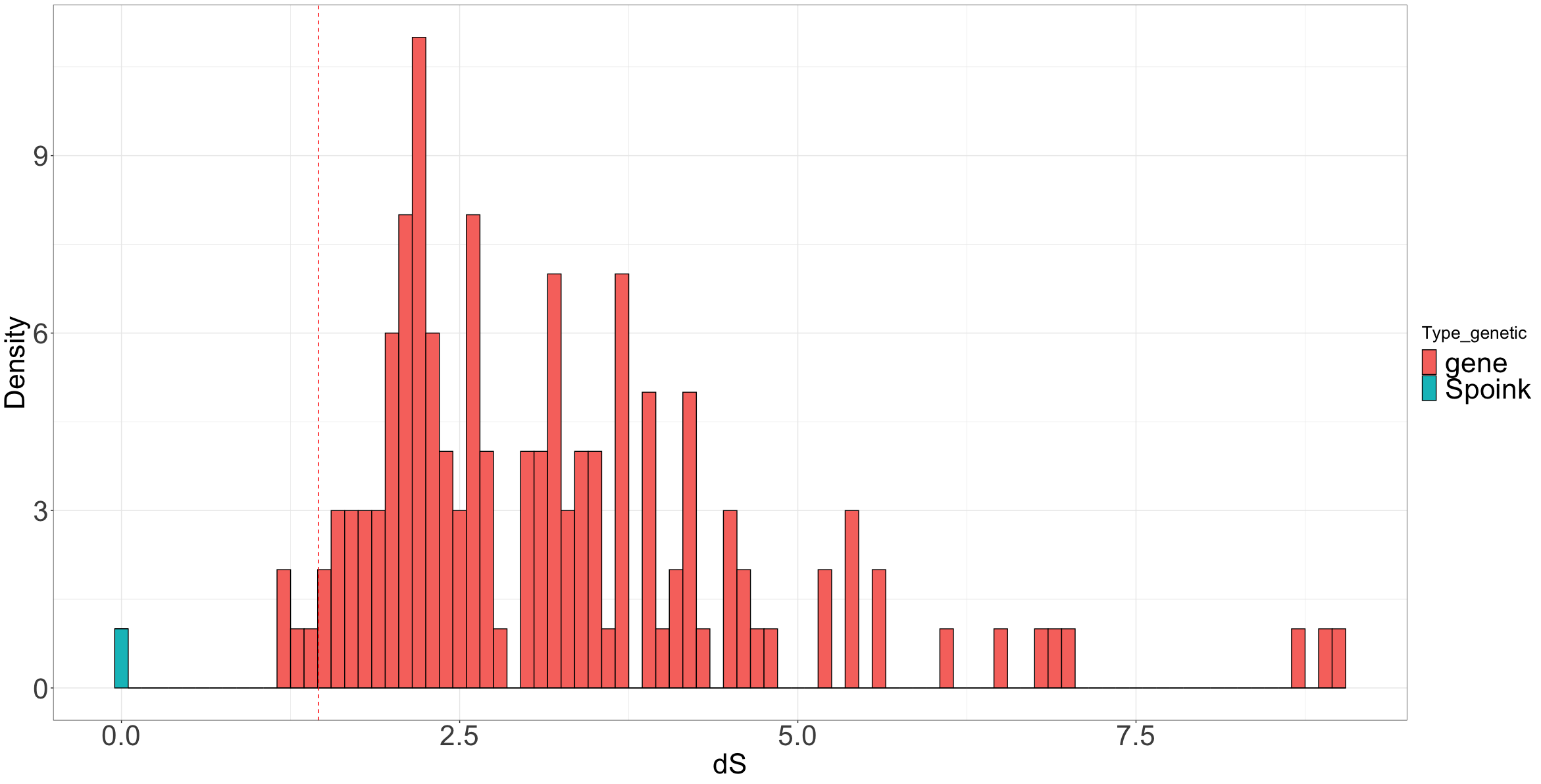

Supplement: S9 Fig — For Spoink we used the shared part of the longest ORF (green). The red dashed line is the 2.5% quantile of nuclear genes [85]. Note that the dS of Spoink is lower than the dS of any of the orthologous genes shared between D. melanogaster and D. willistoni, consistent with a horizontal transfer of Spoink between the two species. The genes were obtained with the software BUSCO [119]. The predicted proteins were aligned using Clustal Omega [120]. The codons information from the protein alignment was used for the nucleotide alignment using PAL2NAL [121]. The dS was calculated using the software PAML. (PNG) [file pgen.1011201.s009.png]

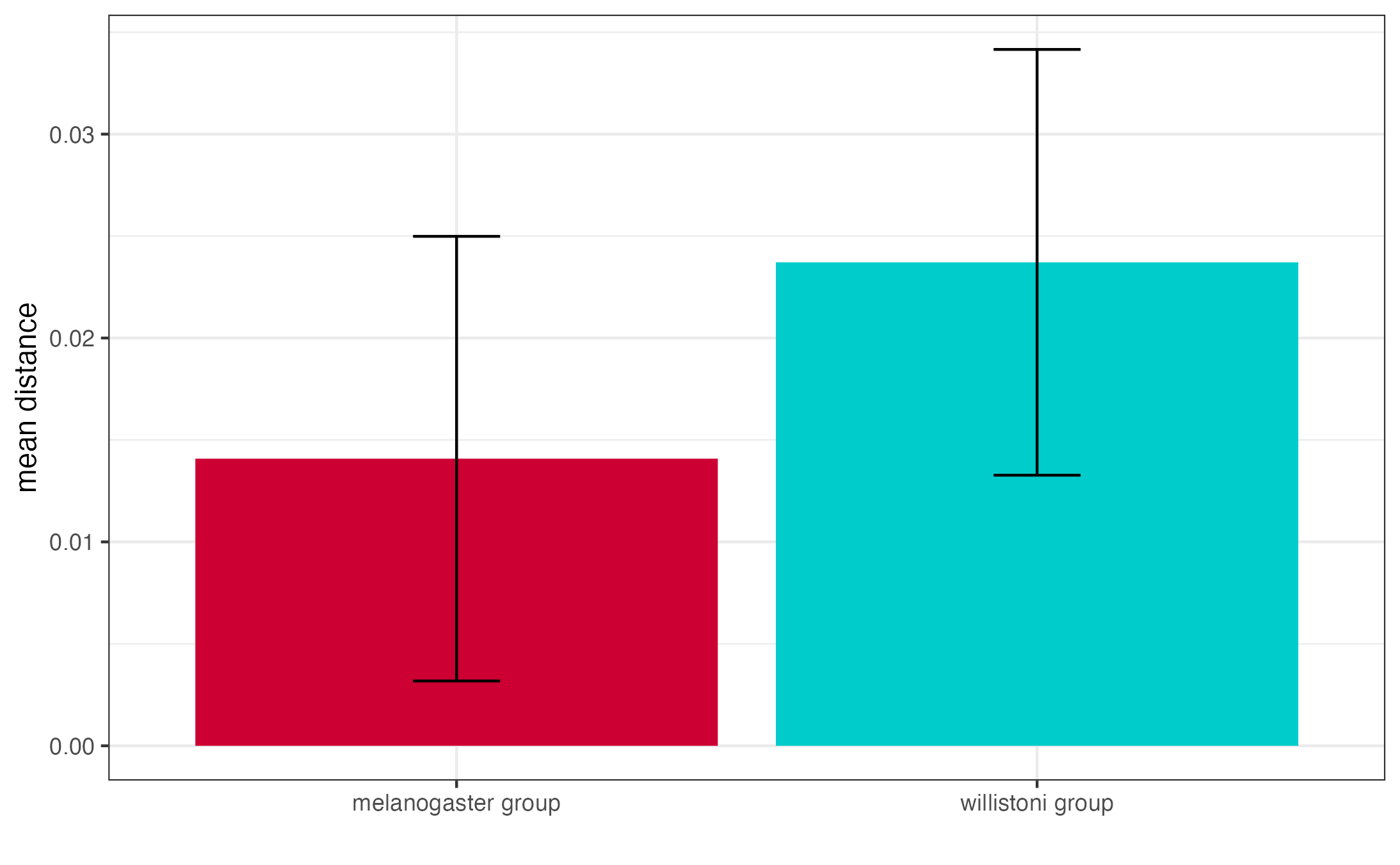

Supplement: S10 Fig — Distances within the willistoni group are significantly longer than the distances in the melanogaster group (t = −6.31, df = 193.88, p = 1.762e − 09). Note that this test accounts for the phylogenetic information of the tree using the distances of the insertions within the two groups. (PNG) [file pgen.1011201.s010.png]
