# Supplementary material for: Spoink, a LTR retrotransposon, invaded D. melanogaster populations in the 1990s
Source: PLoS Genet. 2024 Mar 26;20(3):e1011201. doi: 10.1371/journal.pgen.1011201 (PMC10965091; doi:10.1371/journal.pgen.1011201)
Supplement: Attachment — Submitted filename: Response_to_reviewers.pdf [file pgen.1011201.s020.pdf]

Response to Editor

Dear Dr. Kofler,

Thank you very much for submitting your Research Article entitled 'Spoink, a LTR retrotransposon, invaded D. melanogaster populations in the 1990s' to PLOS Genetics.

The manuscript was fully evaluated at the editorial level and by three independent peer reviewers. The reviewers appreciated the attention to an important topic but identified some concerns that we ask you address in a revised manuscript. In particular I want to draw your attention to the comments of Reviewers 2 and 3 regarding the presence of closely related elements in other Drosophila species (including some apparently not reported in your manuscript) which should be carefully considered in your inference/discussion of the donor species and the evolutionary history of this element in general. These are compulsory points that should be addressed in your revision.

We therefore ask you to modify the manuscript according to the review recommendations. Your revisions should address the specific points made by each reviewer.

We thank the Reviewers and the Editor for the comments. We aimed to address all comments carefully, especially those highlighted by the Editor.

Apart from the edits suggested by the Reviewers we made one additional change to the manuscript. While this work was under review we got notified by Silke Jensen that RepBase has an entry called '*Gypsy-78_DWil*' that has a similarity to *Spoink* (99.7% for internal sequence and 93% for the LTR). As RepBase is behind a Paywall we do not have access to the sequence. This is problematic since we cannot double check this information but Silke Jensen kindly sent us the RepBase entries. This finding has little consequences for our work, apart from the fact that it is in agreement with our suggestion that the donor of *Spoink* may be a species from the *willistoni* group.
For the sake of completeness we are now mentioning the similarity of *Spoink* to this RepBase entry in the manuscript. Furthermore we are now thanking Silke Jensen in the acknowledgments.

With the best regards,
Riccardo Pianezza, Almorò Scarpa, Prakash Narayanan, Sarah Signor, Robert Kofler

Response to Reviewer 1

This study reports a new gypsy-like retrotransposon which has apparently invaded populations of Drosophila melanogaster through recent horizontal transfer taking place

between 1983 and 1993. The source of the transfer may be a Drosophila species belonging to the willistoni group, which would place the transfer somewhere in South America after D. melanogaster reached this continent. I enjoyed reading the paper. It is well-written, with well-crafted, informative figures. I agree with the authors that this is an important and interesting observation that opens many new questions and that will be useful to further study the causes and consequences of horizontal transfer through multiple angles. I am thus overall enthusiastic and would like to congratulate the authors for this work. I would however also like them to take into considerations a few comments.

1 – I am wondering why the search for Spoink in other Drosophila species was limited to long-read assemblies. When performing a blastn search using one Spoink insertion (CM034735.1 6915682 6920905) as query on all Drosophila assemblies available in Genbank I found many insertions >90% identical to this insertion in species not included in fig 5 (gaucha, nebulosa, fulvimacula, leonis, medidiana, meridionalis, anceps) as well as in species in which Spoink is reported to be absent in fig 5 (e.g. in multiple assemblies of D. simulans). Some of these assemblies may have been deposited in Genbank after the study was conducted, but not all. The presence of multiple insertions >99% identical to Spoink in multiple simulans populations is puzzling and I would think it would be pertinent to have a closer look at them and include them in the study. Similarly, I found several copies of Spoink in D. paulistorum that show a higher ID (from 99.07 to 99.5%) to D. melanogaster Spoink than shown in fig 5. This might be due to the fact that I used a copy rather than the consensus but I would like to encourage the authors to further consider these paulistorum copies as they may help refine their scenario of transfer, perhaps suggesting that more than one transfer occurred, including some events more recent than 1993? The paulistorum copies I am referring to are: JAECXG010000750.1 67337 to 71947, JAECWZ010000441.1 146657 to 151263, JAECXG010000663.1 1412470 to 1417089.

We thank the reviewer, Clement Gilbert, for comments and the investigation of *Spoink* in other *Drosophila* species. We aimed to address these comments carefully.

About *D. simulans*. The reviewer is correct, some *D. simulans* assemblies (e.g. SZ129) have Spoink insertions while others (e.g. 14021-0251.006) do not have them.

This suggests that *D. simulans* may also have been recently invaded by *Spoink*. Indeed, we are currently working on a manuscript which is investigating the spread of *Spoink*, and another LTR-retrotransposon (*Shellder*), in several species of the *melanogaster* group (*D. melanogaster* does not have *Shellder*) . For this follow-up we have compiled a data set of *D. simulans* strains collected during the last 70 years. These data suggest that *D. simulans* was invaded by *Spoink* between 1995 and 2015 (i.e. after *D. melanogaster*; see picture below). The important question is how relevant is this recent invasion of *Spoink* (and *Shellder*) in *D. simulans* for the current manuscript.

For the current manuscript we have three main aims:
1) show that *Spoink* invaded *D. melanogaster* between 1983 and 1993
2) characterize *Spoink* (gypsy/mdg3, LTRs, insertion bias etc)
3) show that the ultimate donor of *Spoink* is a species from the *willistoni* group

The presence of *Spoink* in *D. simulans* finding may be relevant for the 3rd point, as it raises the question whether *D. simulans* is the donor of *Spoink* and not a species of the *willistoni* group. In this revised manuscript we provide two lines of evidence that *D. simulans* was also recently invaded by *Spoink* and thus cannot be the ultimate donor.

i) For consistency, we solely relied on the 101 drosophilid genomes in the initial submission (https://elifesciences.org/articles/66405). The assembly of *D. simulans* strain 006 from the 101 genomes does not have Spoink insertions. In the revised manuscript we included an additional *D. simulans* assembly, SZ129, having *Spoink* insertions (https://journals.plos.org/plosgenetics/article?id=10.1371/journal.pgen.1010914). This shows a patchy distribution of *Spoink* within *D. simulans*, where one assembly has Spoink and the other one does not. Such a patchy distribution suggests a recent invasion of *D. simulans* by *Spoink*.

ii) we included *Spoink* insertions from SZ129 into our phylogenetic analysis (Fig 5B), which shows that *Spoink* insertions of *D. simulans* have very short branches and group together with the other recently invaded species (*D. sechellia* and *D. melanogaster*). These short branches suggest that *Spoink* invaded *D. simulans* recently.

iii) we are now mentioning that Spoink likely invaded both species, *D. simulans* and *D. sechellia,* very recently and that we have a manuscript in preparation where the reader can expect further information.

It is however feasible that *Spoink* did not infect *D. melanogaster* directly from a species of the *willistoni* group, but indirectly, e.g.: *D. willistoni* -> *D. simulans* -> *D. melanogaster*.

But this does not have any effect on our main finding that a species of the *willistoni* group is likely the ultimate donor of *Spoink*. In the revised manuscript we are now mentioning in the Discussion that *D. melanogaster* may have gotten *Spoink* indirectly. “It is also feasible that *Spoink* invaded *D. melanogaster* indirectly, for example using *D. simulans* as intermediate host, in which case the *Spoink* invasion in *D. melanogaster* may have been triggered in almost any geographic region (both, *D. simulans* and *D. melanogaster*, are cosmopolitan species (Capy2004)).

“

However, the time-series data used in the follow-up work suggests that *D. simulans* got *Spoink* after *D. melanogaster* (*Dmel* 1983-1993; *Dsim* 1995-2015). See figure from follow-up paper:

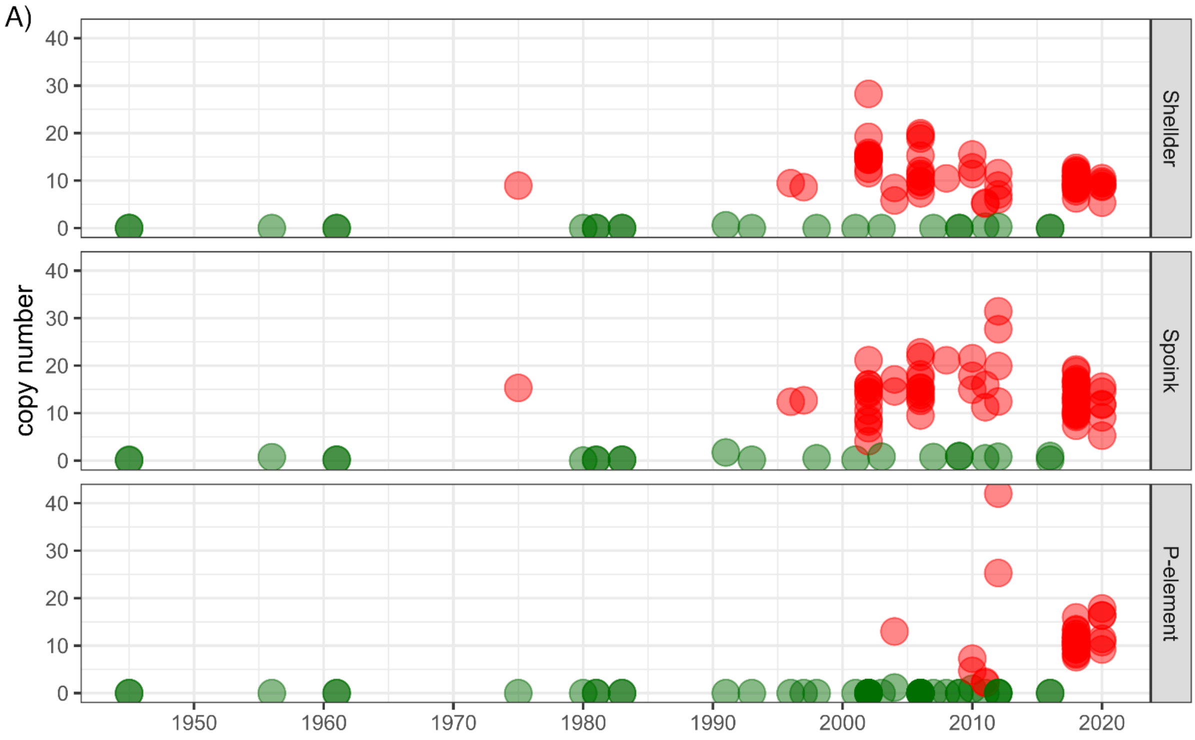

Regarding the long-read assemblies: contigs generated by short-read assemblies usually terminate at repetitive sequences. The short reads (e.g. 150bp or 600bp with some paired-end) cannot span most TE junctions (in the case of Spoink the junctions are >5000bp apart). This problem is especially pronounced for transposons where the dispersed insertions have a very similar sequence such as for *Spoink* (Fig. 5B). Due to these limitations, short-read assemblies may not contain full-length insertions of Spoink, even if such full-length insertions are present in the genome. To avoid positively misleading statements about the absence of Spoink in an assembly (while in fact *Spoink* being present in the genome) we solely focused our efforts on long-read assemblies. To make this more clear we added the following in the revised manuscript: "We did not consider short-read assemblies, as TEs may be incompletely represented in these assemblies (Wierzbicki2020)"

Regarding the species *gaucha*, *nebulosa*, *fulvimacula*, *leonis*, *meridiana*, *meridionalis* and *anceps*. These assemblies were not available when we performed the analysis. We investigated those assemblies to test whether inclusion of these species has an effect on our main message that a species from the *willistoni* group acted as donor of *Spoink*. Indeed, we found *Spoink* insertions in these species. Out of these newly analyzed species insertions of *D. nebulosa* - a species of the willistoni group - are most similar to *Spoink*. But these insertions in *D.nebulosas* have a lower similarity than Spoink insertions in the other species of the willistoni group that are currently shown in the manuscript (blueish colors; see below). *Spoink* insertions of all these newly investigated species are thus more diverged from the *D. melanogaster* Spoink than insertions from the other *willistoni* group species (blueish colors; see below). Most likely therefore, these species could not have served as the donor of *Spoink* in *D. melanogaster* and inclusion of these species would not have an effect on our main messages.

Assemblies of novel (*Drosophila*) species are currently published at an unprecedented rate, especially Kim et al (2023; https://www.biorxiv.org/content/10.1101/2023.10.02.560517v1) released several novel assemblies after the analysis of this work were done. These are fantastic resources, but it also means that there will continuously emerge novel assemblies that have not yet been considered in any analysis. To finish a work, it is necessary to set a threshold. Since these novel assemblies have no effect on our main message, we prefer to set a simple and consistent threshold at the 101 genomes paper (https://elifesciences.org/articles/66405) and to not include these novel assemblies - that still have not passed peer-review - into the paper.

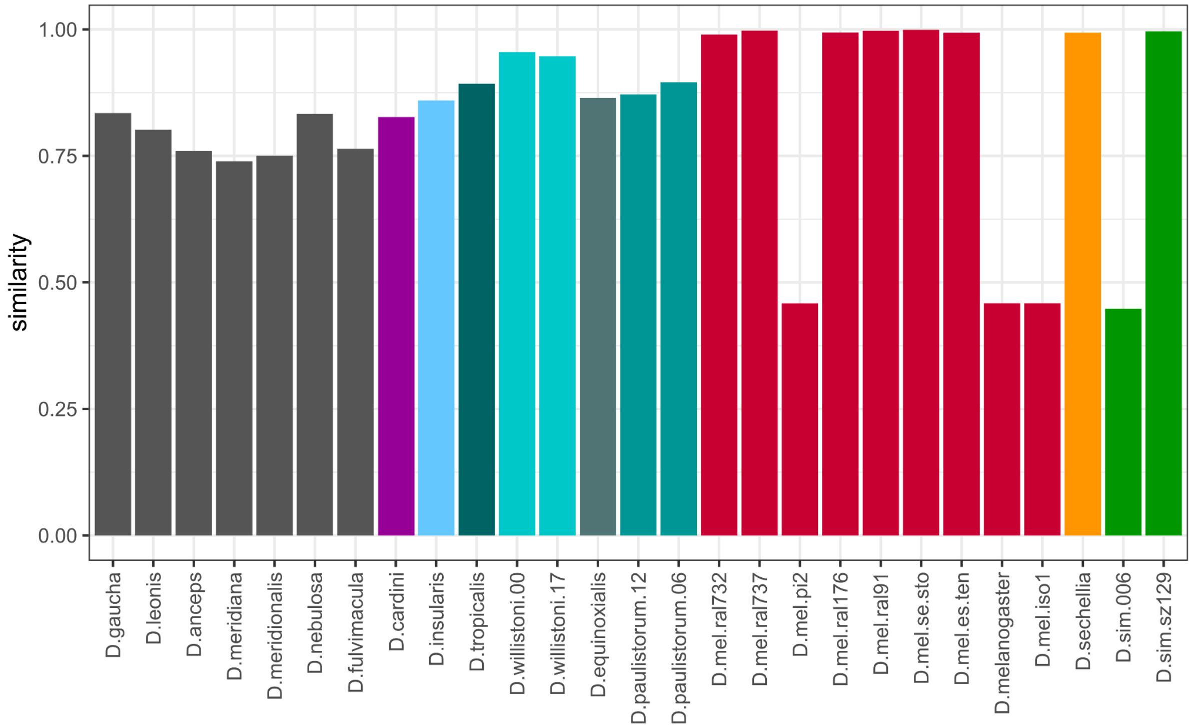

Regarding the three *D. paulistorum* insertions.
We first checked whether they were already present in our analysis. Two insertions, JAECXG010000750.1:67337-71947 and JAECXG010000663.1:1411382-1417089 (corresponding to coordinates 1412470 - 1417089 mentioned by the reviewer), were identified in the assembly L06 and are shown in our phylogenetic tree (Fig. 5B: Dpau-3 and Dpau-5). The other insertion (JAECWZ010000441.1 146657 to 151263) was found in a different assembly (L12: from the 101 Drosophilids assemblies paper: https://doi.org/10.7554/eLife.66405). It was not included in the tree as we opted to use only one assembly per species (otherwise the tree would grow too large). However this insertion was considered for the RepeatMasker analysis shown in figure 5A.

The tree (Fig. 5B) indicates that these *D. paulistorum* (Dpau-3, Dpau-5) insertions form a distinct branch, separate from the Spoink insertions in *D. melanogaster* despite the high sequence similarity noted by the reviewer. We investigated this further. We observed that the coordinates provided by the reviewer did not encompass the full-length *Spoink* element in *D. paulistorum*, but only a subsequence with a high similarity (spanning approximately from base 1 to 4610 out of 5216 bases). By contrast, the *Spoink* insertions considered in our tree

(Dpau-3, Dpau-5) had both LTRs, suggesting that they were full-length insertions (length >5000bp). Hence, the high sequence similarity noted by the reviewer is confined to a subsequence of *Spoink* (from bases 1 to 4610). When the full-length elements are considered (as in our tree), the *Spoink* insertions from *D. paulistorum* and *D. melanogaster* form distinct branches.

Following we show the coordinates of the full-length insertions for the sequences mentioned by the reviewer
-> JAECXG010000750.1:67337-71947 (4610 bp)
= JAECXG010000750.1:67019-71947 (4928 bp; Dpau-5 from assembly L06)

-> JAECWZ010000441.1:146657-151263 (4606 bp)
= JAECWZ010000441.1:146657-151814 (5157 bp; assembly L12)

-> JAECXG010000663.1:1412470-1417089 (4619 bp)
= JAECXG010000663.1:1411382-1417089 (5707 bp; D.pau-3 from assembly L06)

2 – The presence of Spoink in one strain collected in 1979 in Providence (USA) is quickly dismissed as a possible contamination. Is there any other information that may help support this hypothesis? When was this Providence strain sequenced and where? Does it have the P element? Any genomic feature that may help support contamination? Has contamination between Drosophila strains been noticed before?

Yes, this strain has the *P*-element but this is expected, as the *P*-element largely invaded *D. melanogaster* before 1979 (https://www.ncbi.nlm.nih.gov/pmc/articles/PMC393661/)

This strain putatively contaminated with *Spoink* is part of the sequencing effort published in 2021 in MBE (https://doi.org/10.1093/molbev/msab368). The authors state:

"The 65 isofemale lines collected between 1975 and 1983 were originally collected by Dr Margaret Kidwell and had been maintained at the University of Wisconsin by Dr Rayla Temin. These strains had been maintained in vials, in a separate tray from other stocks. Although it is possible for contamination to occur in fly stocks over time (Frochaux et al. 2020), and we cannot be certain that none has occurred in our strains, it seems clear that contamination would need to affect a large fraction of our strains in order to meaningfully impact analyses such as the frequency-based outlier scan described below. No phenotypes consistent with common laboratory mutant stocks (e.g., eye color, body color, wing morphology) were observed in any of them. Additional evidence against contamination of these stocks was obtained from the IBD and PCA described below."

Thus, the authors cannot rule out contamination, but based on a PCA they did not consider contamination likely. It is however worth noting that contamination could lead to the spread of a TE even if contamination did not lead to any introgressed fragments. A TE may spread in the genome even if the genomic-background of the contaminating strain is entirely lost by drift or negative selection. Such contamination events would not be noticeable by PCA, but they would be noticeable by the presence of the TE. The alternative hypothesis would be

that *Spoink* was already present in North America around 1979 but only in very few strains. It remained dormant until worldwide populations got infected before 1993. It would also suggest that North American populations were invaded early by *Spoink*. While possible, we opted to take the more conservative approach, and avoid overinterpreting the presence of *Spoink* in a single strain collected around 1979.

In the revised manuscript, we are now mentioning in the Discussion an alternative hypothesis, i.e. that the presence of *Spoink* in a strain collected in 1979 could be due to the lag-time between the HT event and the world-wide spread of a TE: "The presence of *Spoink* in a strain collected around 1979 in Providence (USA; fig. 2B) could be due to this lag time (or contamination)."

Minor comments listed following the order of the manuscript:
1 – Line 32, "termed piRNAs, which are"? Fixed
2 – Line 77: a few mismatches between which two sequences? Do you mean an imperfect poly-A tract? We mean that the poly-A tract may differ in length by a few bases between insertions. We clarified this in the revised manuscript.
3 – Line 79: providing evalues without mentioning which similarity search was done on which database is not very informative. The 'e-values' were obtained from a BLASTp search of the *Spoink* polyprotein, where the domains were visualized as part of the search result (based on the CDD - Conserved Domain Database). We repeated this analysis by directly searching the *Spoink* polyprotein in the Conserved Domain Database (v3.20; https://academic.oup.com/nar/article/51/D1/D384/6882111?login=true). This slightly changed the results. According to this direct search, *Spoink* has three conserved domains: RT, RNase HI and integrase. In the revised manuscript we are now mentioning that e-values are based on a search in CDD v3.2.
4 – Line 80: RNase H (not HI)? A search of the *Spoink* polyprotein in CDD v3.20 suggests the conserved domain is RNase HI.
5 – Line 81: env (lower case italics) *may* refer to the gene rather than the protein, not sure whether this comply with PLOS Genetics rules. According to FlyBase, gene names should be italicized. https://wiki.flybase.org/wiki/FlyBase:Nomenclature#Italic, In the revised manuscript we italicized *env*
6 – Caption figure 1: use LTR retrotransposon rather than LTR transposon to fit with the core text? Fixed
7 – Line 89: above it is written that the gag pol is typical for ty3/gypsy. Is it typical or atypical and if atypical please elaborate? We considered it atypical since the gag-pol polyprotein is encoded by a single ORF and Eickbush and Malik mentioned no known cases of Ty3/gypsy TE having a polyprotein (https://www.researchgate.net/publication/229067718_Origins_and_Evolution_of_Retrotransposons). We further investigated the literature and discovered that more recent works found gag-pol polyproteins for Ty3/gypsy TEs (Neumann, Pavel, et al ,Mobile DNA 10 (2019), Wells, J. N., & Feschotte, C., Annual review of genetics (2020)). In the revised manuscript we are now stating in the results: "A gag-pol polyprotein as encoded by *Spoink* was observed for some Ty3/gypsy transposons (Neumann, Pavel, et al ,Mobile DNA 10 (2019),

Wells, J. N., & Feschotte, C., Annual review of genetics (2020)) but not for others (Eickbush2002).”
8 – Line 156: collected in 1993 Fixed
9 – Line 179: will then spread, not than Fixed
10 – figure 3: please add a color legend and perhaps mention that the intensity of color in the B panel varies because dots appear superimposed on each other? Also pleas eexplain what are the number in the table in panel A. We added a legend to Fig 3A. Furthermore we explain in the caption that numbers are estimated copy numbers of the TEs and that color intensities of Fig 3B are due to overlapping dots.
11 – Line 205: were further instead of we further Fixed
12 – Fig 5 b: I would personnally much refer to sea a star tree (rather than circular) with branch lengths proportional to inferred distance and a legend for branch lengths. Please consider adding simulans and other paulistorum insertions in this analysis as suggested in my earlier comment. We added *Spoink* insertions found in the *D. simulans* assembly SZ129. The *D. paulistorum* insertions are discussed above. We also added a star tree in the supplement, but we would prefer having the circular tree in the main figure (it's more compact and labels are not overlapping).
13 – Line 261: 15 generations Fixed
14 – Line 267: then Spoink will have Fixed
15 – Line 273: to which extent human activity Fixed
16 – Regarding the possible transfer from willistoni group to melanogaster, are there any ecological feature of melanogaster and willistoni sp that may support close physical contact between these species? Habitat fragmentation caused by human impact extensively brings forest habitat species (like *D. willistoni)* into contact with species from anthropogenic environments (like *D. melanogaster*). Penariol and Ravazzi conducted a study (source: 10.1186/2193-1801-2-114) focused precisely on this scenario involving native and invasive drosophilids. *D. willistoni* is the most common drosophilid in South American forests with a wide distribution (source: https://doi.org/10.1093/oxfordjournals.jhered.a022984). We think that HTs are more likely when two species frequently come into contact, as is likely the case for *D. melanogaster* and *D. willistoni*. We added a brief description of this to the Discussion.
17 – Line 323: piRNA-based Fixed
18 – Line 331: may then compensate Fixed
19 – Line 429: please add a dot at the end of the sentence. Fixed
20 – Please consider submitting Spoink consensus sequence to repbase and other relevant databases. *Spoink*’s LTR and internal sequence have been submitted to RepBase. We also describe the similarity of *Spoink* to ‘*Gypsy-78_DWil*’ (see comment to Editor)

Response to Reviewer 2

This manuscript provides a thorough investigation of a clear case of horizontal transfer of a previously unchacter eized LTR retrotransposon in one of the most well-studied model organisms, D. melanogaster. The evidence provided is rigorous and compelling and I only have relatively minor comments that could improve the work.

General comments

1) It would be helpful if the authors could review the required evidence to demonstrate horizontal transfer of a transposon, and then detail which pieces of evidence presented in the paper correspond to the requirements to demonstrate HTT. Wallau et al. 2012 GBE and Peccoud et al. 2018 Bioessays provide convenient reviews that summarize HTT evidence requirements. Interestingly, the authors also have an opportunity to formally extend the previously considered categories of evidence that support HTT by adding presence/absence across a time series as a new category of evidence (which the authors satisfy in the current work).

Both reviews mention three main lines of evidence i) phylogenetic incongruencies between the TE and the species ii) patchy distribution of TEs among related species and iii) the high similarity) of the TE among related species (i.e. dS of TE is lower than of the vertically inherited host genes).

Importantly all three lines of evidence for recent HTT (horizontal transfer of a TE) are indirect. Although both reviews mention that the recent HT of the *P*-element from *D. willistoni* to *D. melanogaster* is one of the best documented and most unequivocal cases of a HTT, both reviews did not mention the more direct evidence supporting the HTT of the *P*-element, i.e. the absence of the *P*-element in old lab strains and the presence in recently collected strains (Anxolabhere 1988, Kidwell 1983). Perhaps this is not mentioned because the focus of the reviews is to infer HTT from the growing number of publicly available genomes. Another reason may be because such more direct evidence for a HTT is a serendipitous and a rare event that is therefore limited to very few cases of HTT.

We are now discussing the indirect + more direct evidence for the recent HTT of Spoink:

“Horizontal transfer of a TE is usually inferred from three lines of evidence: i) a patchy distribution of the TE among closely related species, ii) a phylogenetic discrepancy between the TE and the host species and iii) a high similarity between the TE of the donor and recipient species, which is frequently quantified by the synonymous divergence of the TE (Peccoud2018,Wallau2012).

All of these three lines of arguments support a horizontal transfer of *Spoink* in *D. melanogaster*, with a species of the willistoni group being the likely donor.

First, we found a patchy distribution among species of the melanogaster group (where for *D. simulans* we even have a patchy distribution among different strains; fig 5A).

Second, *Spoink* insertions of *D. melanogaster* (and other species that may have gotten *Spoink* recently) are nested within species of the willistoni group (fig 5B), a clear phylogenetic discrepancy.

Third, we found that the synonymous divergence of *Spoink* is lower than for all orthologous genes in *D. melanogaster* and *D. wilistoni*.

In addition to this classical but indirect lines of evidence, we have however more direct and thus more compelling evidence for the horizontal transfer of Spoink. Based on strains collected during the last hundred years from all major geographic regions we showed that *Spoink* insertions were absent in all strains collected before 1983 but present in all strains collected after 1993 (using Illumina short read data, long-read assemblies, and PCR/Sanger sequencing). This makes *Spoink* one of the best documented cases of a recent horizontal transfer of a TE, similarly to the *P*-element where strains collected during the last 100 years support the recent horizontal transfer (Kidwell1983, Anxolabehere1988).

2) The authors should discuss the current findings in the context of prior genomic evidence for LTR retrotransposon insertions being young in in D. melanogaster by Bergman and Bensasson 2007 PNAS and comparative genomic evidence by Bartolome et al 2008 Genome Biology, which suggest that widespread HTT of LTR retrotransposons is common in D. melanogaster and has been an ongoing process for longer than the last 200 years.

In the revised manuscript we are now mentioning in the Discussion: “ It is possible that the high rate of TE invasions continues beyond the past 200 years since many LTR transposons in *D. melanogaster* are likely of very recent origin (possibly <16.000years (Bergman2007,Bartolome2009)).”.

3) The authors make the claim that it is surprising that HTT of Spoink went unnoticed because D. melanogaster is investigated heavily at the genetic level. However, this is not surprising to me because only a limited number of lab strains of D. melanogaster strains were intensively studied for the last 100 years, and complete genomes of natural populations have only been investigated very recently.

The finding that *Spoink* invaded *D. melanogaster* was surprising to us. We did not expect it and solely found it by coincidence when investigating the TE composition of other species. However, the reviewer is correct in that many studies focused on older lab strains. We therefore toned down the statement mentioning that “it is **perhaps** surprising”.

4) The authors often compare results from Spoink to the P-element. However, the P-element is arguable not the best control to contrast with distribution and variation in Spoink since it is a DNA transposon, not an LTR retrotransposon. I would urge the authors to add control analyses to the main or supplemental text comparing results for Spoink to another Ty3/gypsy LTR retrotransposon.

The *P*-element does not serve as a control, we included it as a reference for dating the invasion. In particular one main message is that *Spoink* invaded after the *P*-element, therefore it was necessary to show the timing of the *P*-element and the *Spoink* invasion (e.g. Fig 3A, 3B). For the insertion preference of *Spoink* we just included the *P*-element to show that preferences may differ among TEs, it was actually not necessary to show the *P*-element in this graph.

Specific comments

Line 32: change “are cognate” -to“that are cognate” Fixed
Line 79: change “e-value e=” to “e-value” Fixed
Lines 79, 80, 81: change “e =” to “e-value =” Fixed
Line 89: explain how gag-pol polyprotein for Spoink is atypical for Ty3/gypsy elements. Please see response to similar comment by reviewer 1.

Line 107: change “of single copy” to “a sample of single copy” Fixed
Line 136: change “present in all” to “present in centric heterochromatic regions of all” we changed it to “are present in heterochromatic regions of the centromeres of all”
Line 155-6: Is there any other genome-wide evidence that the Providence 1979 strain is contaminated? Unfortunately no, we however think contamination is the more conservative interpretation. In the revised discussion we are now offering an alternative explanation. For more details please see response to Reviewer 1.
Line 172: change “Spoink composition” to “Spoink sequence variation” Fixed
Line 181-2: cite or provide evidence for Blood and Opus being different in Zimbabwe populations Done. We are now citing our previous work (Scarpa et al 2023). Also we spotted a mistake, we replaced “*Blood*” with “*412*” (412 shows a geographic heterogeneous pattern not *Blood*)
Line 205: change “we further” to “were” Fixed
Line 224: change “we found that Spoink is silenced” to “we found evidence that Spoink is silenced” Fixed
Line 301: change “identified TE invasion in D. melanogaster during the last” to “TE invasion in D. melanogaster that has occurred during the last” Fixed.
Line 351: change “Characterisation” to “Structure and Classification” Fixed
Line 385: which version of the TE database from Quesneville was used in the current analysis? Was this obtained from flybase or https://github.com/bergmanlab/drosophila-transposons? We used v9.44; We are now mentioning this in the M&M.
Line 402-3 should “read” and “reads” be replaced with “alignment” and “alignments” since according to my understanding flanking sequences are being aligned not reads. Yes, flanking sequences are aligned. We thus replaced it with “flanking sequences”.
Figure 1: Increase font size of sequence and labels in Fig1A and node support values in Fig 1B. Detail how node support values in Fig 1B were calculated in legend. Provide contig IDs and coordinates of Spoink insertions in Fig 1A.
We increased the font size of Fig. 1 and of the node support in Fig 1B. The new supplementary table 3, provides coordinates of the Spoink insertions used in Fig 1A.
Figure 5: Detail how node support values in Fig 5B were calculated in legend. Node support values are the posterior probabilities estimated by BEAST under standard parameters (Gamma site model, substitution model JC69, mean clock rate = 1.0, strict clock, Yule model). We added a brief description to the caption.
References: Genus names and abbreviations are not capitalized in several references.. Fixed

Response to Reviewer 3

The manuscript titled “Spoink, a LTR retrotransposon, invaded D. melanogaster 2 populations in the 1990s” by Pianezza and Scarpta et al, 2023 reports discovery of a novel TE subfamily and reconstruction of its evolutionary history. This manuscript presents analysis from genomic and piRNA libraries to describe this transposable element termed Spoink and its invasion of D. melanogaster populations three decades ago. Some parts of the manuscript contain rigorous analysis and reasonable inferences but the analysis in other parts is superficial and lacks sufficient details. I have my major and minor concerns on the latter listed below.

Major points:

1. While the evolutionary and population history of Spoink presented is extensive, authors have not performed definitive analyses to statistically test their claim of origin of Spoink from D. willistoni using synonymous substitution changes between single copy orthogous genes and Spoink. This is especially necessary because D. sechelia also carries many Spoink insertions that are more similar to that of D. melanogaster. It seems more likely that Spoink invaded melanogaster subgroup from willistoni group, where melanogaster sp and sechelia sp both incurred this invasion independently from willistoni or Spoink invaded one of the two species in melanogaster subgroup and other acquired it from the former also through horizontal transfer. A dS test of TEs and orthologous genes between species can help distinguish these possibilities.

That a species from the *willistoni* group is the ultimate donor of *Spoink* is supported by the phylogenetic tree (Fig 5B) where insertions of *D. melanogaster*, *D. simulans* and *D. sechellia* are nested within insertions of species from the *willistoni* group. In the revised manuscript we performed the dS test as suggested by the reviewer based on 140 BUSCO genes being orthologous between *D. melanogaster* and *D. willistoni*. dS of *Spoink* was substantially lower than the dS of any of the 140 orthologous genes. We added this information in novel supplementary figure 9.

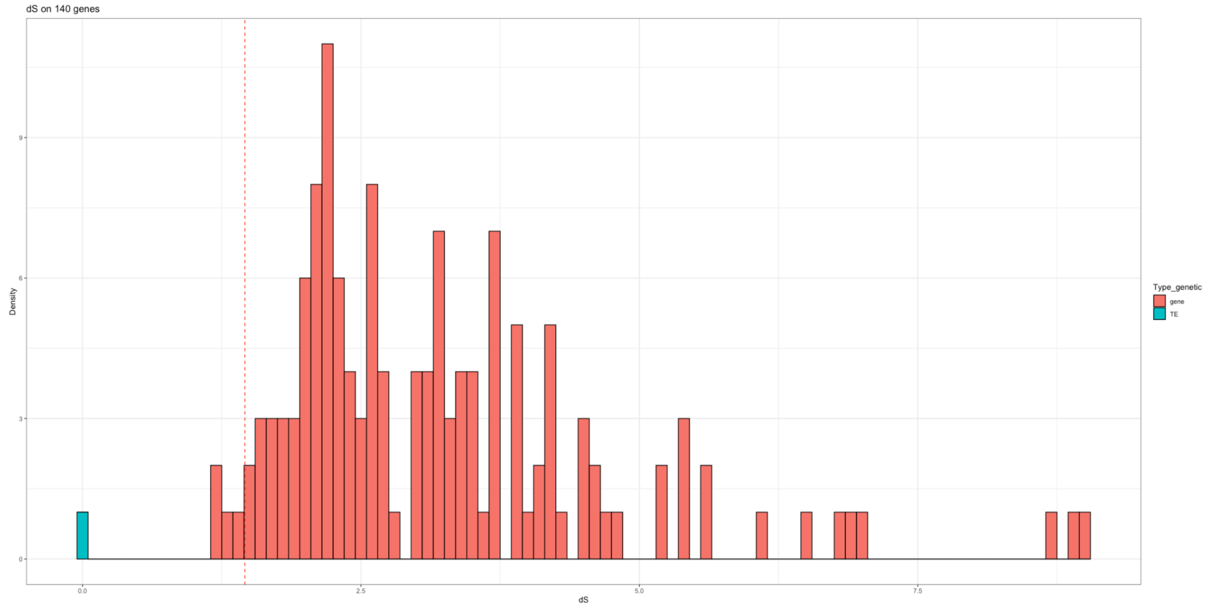

2. Lines 282-83 – “In addition, phylogenetic tree of Spoink has deep branches within the willistoni group, suggesting that Spoink is ancestral in this group.” There needs to be a quantification and test of the branch lengths differences of insertions.

In the revised manuscript, we randomly sampled 100 pairs of branches within two groups i) *Dmel*/*Dsim*/*Dsec* and ii) *willistoni* species. Next we computed the distance for each of these pairs and compared the distances between two groups (willistoni vs Dmel/Dsim/Dsec) with a t-test. This shows that branches within the *willistoni* group are significantly longer than branches in *Dmel*/*Dsim*/*Dsec* group ($t = -6.3194$, $df = 193.88$, p-value = 1.762e-09). Note that this test accounts for the phylogenetic information of the tree using the distances of the insertions within the two groups.

3. Many Figures and Table contain overlapping data points hard to see and text labels extremely small. It will help readers to understand the findings if authors tidy up the figures. For example, data points in Figure 2B are too big and many are overlapping which makes it hard to distinguish whether we’re looking at one or multiple insertions. In figure 5B, some shades of green are very hard to read, especially D. equinoxialis. In figure 2D, shades of yellow make it impossible to distinguish insertions. Figure 3A table and 3B X-axis labels are very small. Figure 3A table, legend lacks any mention of what do the values represent.

We reduced the point size in Fig 2B and 2D. Furthermore we decreased the alpha value (i.e. the transparency) to facilitate distinguishing overlapping insertions. In Fig 2D we switched to dark-green instead of yellow. We also increased the text size in Fig 3A, Fig 5 and Fig 1.

In Fig 5B, the shades are on purpose similar to indicate the related species of the *willistoni* group. However the labels unambiguously indicate the species, for example D.equ indicates *D. equinoxialis*

Minor points:

Lines 219-221 – Authors mention that Spoink insertions in piRNA clusters were quantified but Figure/Data from the Supplement is not referenced. Each Figure/Data should be cross referenced.

We thank the reviewer for spotting this omission. We are now referring to supplementary table 7.

Figure 4 and legend – If the authors are referring to the LTR element HMS Beagle (described and named in Snyder et al, 1982), name should be corrected. Fixed

Figure 2D – Y-axis is labelled "%" but I think the the values are in proportion. Either the values or the axis title should be changed. Same issue in Figure 5A.

The reviewer is correct, Fig 2D shows the proportion. We removed the % label in Fig 2D. The y-axis of Fig 5A shows the similarity expressed as a ratio of RepeatMasker scores (for details see M&M) and does not have a %-label.

Line 43 – "P" of P-element is italicized everywhere except here. Fixed

Line 330 – In use of word "regular" here, I think authors need to be more specific. Do they mean non- piRNA cluster insertions or non-paramutated insertions?

Yes, we mean normal *Spoink* insertions not producing any piRNAs, ie. non-paramuated *Spoink* insertions. We are now mentioning this in brackets.

Line 331 – 'than' needs to be changed to 'then' Fixed

Line 294 – Reference needed. Why D. pseudoobscura or D. persimilis? D. willistoni is a more likely candidate for Spoink and P-element.

We agree with the reviewer that a species from the *willistoni* group likely acted as donor of *Spoink* and the *P*-element. In this section we are discussing why species of the *willistoni* group and not any other species abundantly occurring in the Americas (such as *D. pseudoobscura* and *D. persimilis*) acted as donor of a horizontal transfer of a TE in *D. melanogaster.*

To make this more clear we added the following to the discussion "Why is again a species of the *willistoni* group and not one of these other species acting as donor of a novel TE?" Furthermore, we added a reference for the geographic distribution of these two species.

Lines 296-300 – Again, reference needed.
In this paragraph we are proposing several novel hypotheses that could explain why species of the *willistoni* group are frequently acting as donors of novel TEs in *D. melanogaster*. Since these are our novel hypotheses no references can be provided. To make this more clear we are now mentioning that these are "hypotheses".
