# Supplementary material for: Spoink, a LTR retrotransposon, invaded D. melanogaster populations in the 1990s
Source: PLoS Genet. 2024 Mar 26;20(3):e1011201. doi: 10.1371/journal.pgen.1011201 (PMC10965091; doi:10.1371/journal.pgen.1011201)
Supplement: Attachment — Submitted filename: Spoink_review_comments.docx [file pgen.1011201.s019.docx]

The manuscript titled “Spoink, a LTR retrotransposon, invaded *D. melanogaster* 2 populations in the 1990s” by Pianezza and Scarpta et al, 2023 reports discovery of a novel TE subfamily and reconstruction of its evolutionary history. This manuscript presents analysis from genomic and piRNA libraries to describe this transposable element termed *Spoink* and its invasion of *D. melanogaster* populations three decades ago. Some parts of the manuscript contain rigorous analysis and reasonable inferences but the analysis in other parts is superficial and lacks sufficient details. I have my major and minor concerns on the latter listed below.

Major points:

1. While the evolutionary and population history of Spoink presented is extensive, authors have not performed definitive analyses to statistically test their claim of origin of Spoink from *D. willistoni* using synonymous substitution changes between single copy orthogous genes and Spoink. This is especially necessary because *D. sechelia* also carries many Spoink insertions that are more similar to that of *D. melanogaster.* It seems more likely that Spoink invaded melanogaster subgroup from willistoni group, where melanogaster sp and sechelia sp both incurred this invasion independently from willistoni or Spoink invaded one of the two species in melanogaster subgroup and other acquired it from the former also through horizontal transfer. A dS test of TEs and orthologous genes between species can help distinguish these possibilities.
2. Lines 282-83 – “In addition, phylogenetic tree of Spoink has deep branches within the *willistoni* group, suggesting that Spoink is ancestral in this group.” There needs to be a quantification and test of the branch lengths differences of insertions.
3. Many Figures and Table contain overlapping data points hard to see and text labels extremely small. It will help readers to understand the findings if authors tidy up the figures. For example, data points in Figure 2B are too big and many are overlapping which makes it hard to distinguish whether we’re looking at one or multiple insertions. In figure 5B, some shades of green are very hard to read, especially *D. equinoxialis*. In figure 2D, shades of yellow make it impossible to distinguish insertions. Figure 3A table and 3B X-axis labels are very small. Figure 3A table, legend lacks any mention of what do the values represent.

Minor points:

Lines 219-221 – Authors mention that Spoink insertions in piRNA clusters were quantified but Figure/Data from the Supplement is not referenced. Each Figure/Data should be cross referenced.

Figure 4 and legend – If the authors are referring to the LTR element HMS Beagle (described and named in Snyder et al, 1982), name should be corrected.

Figure 2D – Y-axis is labelled “%” but I think the the values are in proportion. Either the values or the axis title should be changed. Same issue in Figure 5A.

Line 43 – “P” of *P*-element is italicized everywhere except here.

Line 330 – In use of word “regular” here, I think authors need to be more specific. Do they mean non-piRNA cluster insertions or non-paramutated insertions?

Line 331 – ‘than’ needs to be changed to ‘then’

Line 294 – Reference needed. Why *D. pseudoobscura* or *D. persimilis*? *D.* *willistoni* is a more likely candidate for *Spoink* and *P-element*.

Lines 296-300 – Again, reference needed.
